# One drug to treat them all: ethical implications of the MORDOR trial of mass antibiotic administration to reduce child mortality

**DOI:** 10.7189/jogh.09.010305

**Published:** 2019-06

**Authors:** Clarence C Tam, Vittoria Offeddu, Jane Mingjie Lim, Teck Chuan Voo

**Affiliations:** 1 Saw Swee Hock School of Public Health, National University of Singapore and National University Health System, Singapore; 2 London School of Hygiene & Tropical Medicine, London, United Kingdom; 3 Centre for Biomedical Ethics, Yong Loo Lin School of Medicine, National University of Singapore and National University Health System, Singapore

The recent Macrolides Oraux pour Réduire les Décès avec un Oeil sur la Résistance (MORDOR) cluster-randomised trial of mass azithromycin administration in Malawi, Niger and Tanzania demonstrated a 13.5% overall reduction in under-5 childhood mortality among communities receiving twice-yearly azithromycin [1]. Subgroup analyses suggested that these mortality reductions were consistent only in children <6 months, and that overall results were mainly driven by mortality reductions in Niger, where both mortality and mortality reductions were considerably greater than in the other two countries. Uncertainty in effect estimates from Malawi and Tanzania was high, because baseline mortality in these two countries was much lower than assumed in the trial design.

Further evidence will be needed to determine whether this is a generalisable finding and how much of an impact mass azithromycin administration can have on infant mortality. However, at a time of concerted efforts to integrate reductions in both infant mortality and antimicrobial resistance as part of the Sustainable Development Goals, this trial highlights the need to deal with complex dilemmas in global health, and raises important ethical issues about rational antibiotic use. As the authors of the trial point out, any use of mass antibiotic treatment in this way would need consideration of the potential adverse effects of administering azithromycin to young children. These could result from both adverse drug reactions and selection of antibiotic-resistant bacterial strains that could have subsequent clinical implications. Additionally, in public health terms non-maleficence considerations would extend to potential harms not just to azithromycin recipients, but also to wider society. These could include the potential transmission of antibiotic-resistant bacterial strains to others, as well as diminished effectiveness of azithromycin for treating infections caused by these resistant bacteria. The MORDOR trial did not identify serious adverse events attributable to azithromycin among recipients. The trial authors did not specifically report changes in antibiotic resistance in bacterial species in the study population (this work is ongoing), although previous studies have reported increases in carriage of macrolide-resistant *Streptococcus pneumoniae* and *Escherichia coli* in children following mass azithromycin administration for trachoma control [2-6]. Thus, although there is good reason to believe that mass azithromycin treatment would lead to increased levels of bacterial resistance, the clinical implications and public health impact are currently unknown.

Mass administration of azithromycin is recommended by the World Health Organization (WHO) as part of strategies to eradicate both trachoma and yaws [7,8]. The findings of the MORDOR trial could provide a rationale for extending mass azithromycin administration to other settings without endemic trachoma and yaws, as an intervention to reduce general childhood mortality. In many settings where mass antibiotic administration to reduce childhood mortality might be used, general access to antibiotics to treat infections may be limited because of lack of health care infrastructure or resources. Conversely, in many regions of the world over-use and misuse of antibiotics with no clear clinical benefit is common. It could be argued that such inequities in antibiotic access provide reasons to favour use of mass administration to reduce childhood mortality on the basis of social justice. We do not find such arguments convincing, however, as sub-optimal use of antibiotics in one context, whether through over-use or lack of access, cannot be used to justify its questionable use in other contexts. Considerations of equity must be weighed up against issues of beneficence and harm. This raises a fundamental question:

“Given a proven preventive effect of mass azithromycin administration on childhood mortality, what is the moral obligation to provide such treatment (or what are the ethical implications of withholding such treatment)?”

From a global health ethics viewpoint, various perspectives could be used to consider the moral grounds of an obligation to benefit children by means of mass antibiotic treatment [9,10]. We focus on two key perspectives: humanitarianism and human rights. These are often linked in practice but are conceptually distinct. Global health initiatives such as the Global Fund to Fight AIDS, Tuberculosis and Malaria and Gavi, the Vaccine Alliance, are underpinned by a humanitarian perspective [11-13]. Some of the global elimination programmes rely on drug donations, including azithromycin for trachoma control (under The International Trachoma Initiative). At face value, humanitarianism could also be seen as a basis for provision of the same antibiotic en masse to prevent childhood mortality. However, what makes a humanitarian perspective less compelling in this context is that there is no duty of easy rescue: while lives might be saved by this intervention, we might be sacrificing something of comparable moral importance in the form of future disease burden and mortality from increases in antimicrobial resistance.

From a rights perspective, it seems intuitive to consider the extension of life (or the avoidance of preventable death) to be a basic moral and human right. However, this stance in itself does not determine by what specific means this right should be protected. Many childhood interventions are not implemented in certain settings despite proven benefit. Examples include certain vaccines that are under-used because they are considered too costly, because of competing health priorities, or because of technical challenges in implementation. High cost of vaccine and vaccine delivery, for example, are major factors in the slow implementation of sustainable immunisation programmes against rotavirus [14] and human papillomavirus [15,16]. Vaccines against typhoid in have been recommended by WHO for endemic and epidemic settings for over 10 years, but widespread implementation in many settings is hampered by the need to fund other vaccination programmes, as well as lack of specific diagnostic and surveillance infrastructure to monitor disease burden and assess potential vaccine impact [17].

**Figure:**
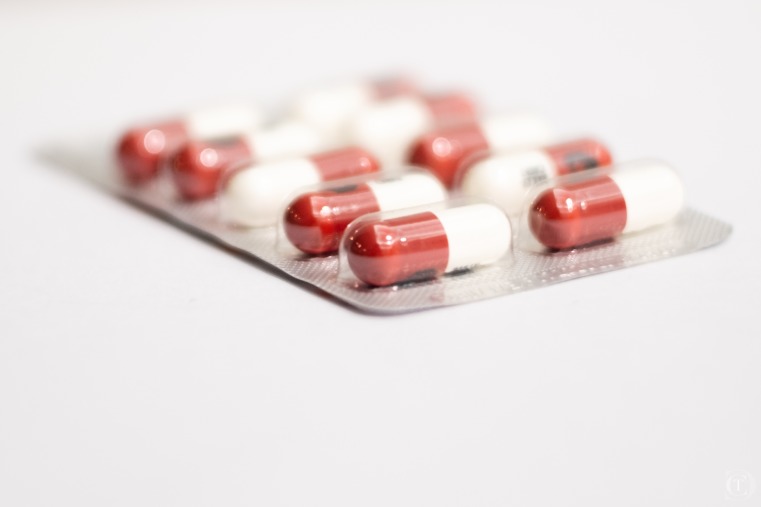
Photo: from the collection of Dr Clarence C Tam (used with permission)

However, a number of key international documents support a human rights argument for mass antibiotic treatment to prevent childhood mortality. The Sustainable Development Goals include targets *“to reduce neonatal mortality to at least as low as 12 per 1,000 live births and under-5 mortality to at least as low as 25 per 1,000 live births”* by 2030. Although these are aspirational targets, they resonate strongly with the international human rights system or framework. Strictly speaking, ‘human rights’ refer to rights stipulated in the Universal Declaration of Human Rights and in human rights treaties such as the International Covenant on Economic, Social, and Cultural Rights (ICESCR). The latter are legally binding on states that have signed and ratified them [18] and may be legally protected and enforced through national constitutions and law [19]. Article 12 of the ICESCR states that *“The States Parties… recognize the right of everyone to the enjoyment of the highest attainable standard of physical and mental health”*, and that steps to be taken progressively (in recognition of resource constraints) to *“achieve the full realization of this right shall include those necessary [...] for the reduction of […] infant mortality and for the healthy development of the child”* [20]. Article 6 of The United Nations Convention on the Rights of the Child [21] spells out every child’s inherent right to life and the duty of states to ensure to the maximum extent possible their survival and development. More specifically, Article 24 includes the following relevant provisions:

- To diminish infant and child mortality;
- To ensure the provision of necessary medical assistance and health care to all children with emphasis on the development of primary health care;
- To combat disease and malnutrition, including within the framework of primary health care, through, *inter alia*, the application of readily available technology and through the provision of adequate nutritious foods and clean drinking-water, taking into consideration the dangers and risks of environmental pollution

To the extent that mass antibiotic administration is a readily available technology that turns out to have a scientifically demonstrable effect in preventing childhood mortality, the international human rights framework seems to confer at least a *prima facie* duty to provide children access to that technology.

The translation of international treaty obligations into rights-based public health programmes should, however, include mechanisms for monitoring and assuring equitable access as well as transparency and accountability, with emphasis on protecting the rights of the most vulnerable and disadvantaged [22,23]. If mass antibiotic treatment for mortality prevention is indeed an obligation correlative to respecting the rights of children, then the question remains of how to deal with the potential adverse consequences.

Here we view the precautionary principle to be particularly pertinent. The precautionary principle asserts that given the potential for serious harm to humans or the environment from a given activity, the burden of proof rests on assuring the safety of the activity, and that preventive measures to mitigate potentially harmful effects of the activity should not be delayed even if uncertainty exists about the probability, severity, remediability or reversibility of harm from the activity. Although commonly used to mitigate potential harms from industry or government activity, particularly in the area of environmental pollution, the precautionary principle applies equally to public health interventions [24]. The UN Declaration on Antimicrobial Resistance recognises that *“within the broader context of antimicrobial resistance, resistance to antibiotics [...] is the greatest and most urgent global risk, requiring increased attention and coherence at the international, national and regional levels”* [25]. In this context, the potential for mass antibiotic administration to drive the emergence and spread of antibiotic resistance constitutes a serious, but uncertain threat to human health. Potential harms from mass antibiotic administration could include future reversals in mortality reduction resulting from increased antibiotic resistance, potential harms to others (including other species) from spread of antibiotic-resistant bacteria, and environmental contamination with antibiotic residues that could drive further resistance. Further, certain antibiotics may be critical medicines in the treatment of specific infections, and this crucial role should be protected – azithromycin, for example, is a key treatment for gonorrhoea. Additionally, the key role that azithromycin plays in health care is underlined by its inclusion in the World Health Organization's Essential Medicines list for the treatment of genital tract infections and trachoma [26].

The precautionary principle asserts that preventive measures should be put in place to mitigate these potential harms. In our view these include mechanisms to monitor and assure the safety of mass antibiotic administration, both in terms of adverse drug reactions and emergence of antibiotic resistance, as well as measures to reduce dependence on mass antibiotic administration, including improvements in sanitation, water quality, immunisation coverage and provision of adequate health care. In addition, these measures should be in place and have the capacity to assure safety from the outset, as the burden of proof must rest on assuring the safety of a planned activity, rather than demonstrating substantial harm from an established activity [24].

We argue that any future implementation of mass azithromycin administration to reduce mortality through as yet undetermined mechanisms should only occur alongside adequate implementation of surveillance mechanisms to monitor adverse drug reactions as well as emergence of antibiotic resistance, with a clear framework for deciding if and when this intervention does not satisfy acceptable levels of safety. In addition, such implementation should occur alongside investment in sanitation and health care infrastructure to reduce reliance on mass antibiotic administration in these communities. Such an implementation recognises that the burden of proof may change over time with accumulating evidence of benefits and externalities. In addition, investment in monitoring systems and alternative infrastructure respects the principle of self-determination, providing communities with increased autonomy to make informed decisions about whether continued dependence on mass antibiotic administration is acceptable. Without such infrastructure in place, the only measurable outcome is mortality, which at least in the short-term always favours mass antibiotic administration. Ensuring the capacity to measure the benefits of mass administration relative to other interventions, and weigh up its benefits against measurable harms, provides the means to make more informed and sustainable policy choices.

The potential to prevent childhood mortality with a single pill – the one drug to treat them all – is a tempting prospect. The road to MORDOR, however, is fraught with pitfalls. A global ethics perspective, though often overlooked, highlights the complex challenges posed by antimicrobial resistance and the need for integrated, cross-sectoral approaches to infectious disease control, global antimicrobial stewardship, sanitation and universal health care, while providing a framework for action that respects the rights of both individuals and the global community.
